# Age independent survival benefit for patients with small hepatocellular carcinoma undergoing percutaneous cryoablation: A propensity scores matching study

**DOI:** 10.3389/fonc.2023.1072054

**Published:** 2023-01-20

**Authors:** Huixin Zhang, Muoyan Xu, Jiashen Shao, Huifang Kong, Xudong Gao, Wei Zhang, Xiujuan Chang, Bin Yang, Yan Chen, Zheng Dong, Jiagan Huang, Zhen Zeng, Yongping Yang

**Affiliations:** ^1^ Department of Liver Diseases, The Fifth Medical Center, Chinese PLA General Hospital, Beijing, China; ^2^ Healthcare Office of Chinese PLA General Hospital, Beijing, China; ^3^ Department of Orthopedics, Beijing Friendship Hospital, Capital Medical University, Beijing, China

**Keywords:** (LTP) local tumor progression, (OS) overall survival, (TFS) tumor-free survival, (HCC) hepatocellular carcinoma, (MRI) magnetic resonance imaging, (AFP) α-fetoprotein, (MWA) microwave ablation, (RFA) radiofrequency ablation

## Abstract

**Background:**

Hepatocellular carcinoma (HCC) is the major cause of malignancy-related deaths worldwide, and its incidence is likely to increase in the future as life expectancy increases. Therefore, the management of elderly patients with HCC has become a global issue. Aim of this study was to assess whether elderly patients with small HCC could obtain survival benefit from cryoablation (CRYO) in a real-world.

**Materials and methods:**

From July 2007 to June 2013, 185 patients with small HCC who underwent curative-intent percutaneous CRYO. All patients were divided into three groups according to age distribution. Overall survival (OS) and tumor-free survival (TFS) were compared between among of groups before and after the 1:1 propensity score matching, respectively. Univariate and multivariate Cox analyses were performed to determine the potential relationships between variables and prognostic outcomes.

**Results:**

One hundred and eighty-five patients (144 men, 41 women) received CRYO for small HCC, including 59 patients with age <50 years, 105 patients with age between 50 and 65 years, and 21 patients with age >65 years. The three age groups showed significant differences in the terms of underlying chronic liver disease and the number of patients with minor postoperative complications. After propensity score matching, the younger and elderly groups showed significant differences in mean OS (P=0.008) and tumor progression (P=0.050). However, no significant differences were shown in mean progression-free survival (PFS) (P=0.303). The Cox multivariate analysis showed that the Child-Pugh grade (HR=3.1, P<0.001), albumin (HR=0.85, P=0.004) and total of bilirubin (HR=1, P=0.024) were the independent prognostic factor for mean OS.

**Conclusion:**

Our propensity-score-matched study suggested that elderly patients with small HCC can achieve acceptable prognostic outcomes with PFS similar to those of younger patients with small HCC after treatment with CRYO, while Child-Pugh grade, bilirubin and serum albumin levels were associated with the prognosis of small HCCs.

## Introduction

Hepatocellular carcinoma (HCC) is one of the most common malignancies and is the third leading cause of malignancy-related deaths (1). HCC is common with increasing life expectancy and is expected to become more common in elderly patients over time. As consequence, the management of elderly HCC patients is now a global issue. Currently diagnostic techniques advances and the availability of screening for high-risk individuals, more and more patients with HCC are being detected at earlier stages, leading to access to radical treatment options, including hepatic resection, liver transplantation and ablation (2). Among them, ablative therapy, especially cryoablation (CRYO) and microwave ablation (MWA), has been considered an effective modality for the treatment of early- and very early-stage HCC (3–5). Compared with open surgery, these treatment modalities have the advantages of being minimally invasive, safe, with fewer complications and faster postoperative recovery.

Previous studies have found that some changes in liver structure and function occur in the elderly population (6–9). In clinical practice, elderly patients with HCC usually have worse liver function and a higher incidence of comorbidities (10). Therefore, the choice of treatment for these patients is often more cautious and the indications for surgery are more stringent. Currently, there are no clear guidelines or strategies to instruct the treatment protocols for elderly patients with small HCC. In particular, it is unclear whether CRYO performed on elderly patients can achieve similar clinical outcomes as younger patients. Therefore, we designed this study in order to clarify the efficacy of the elderly small HCC population after CRYO treatment, as well as to explore the impact of age on clinical outcome. We expect that the findings of this study will contribute to refining the indications for percutaneous ablation in the elderly population and provide an important reference for clinical decision making.

## Materials and methods

### Patients

**Figure 1:**
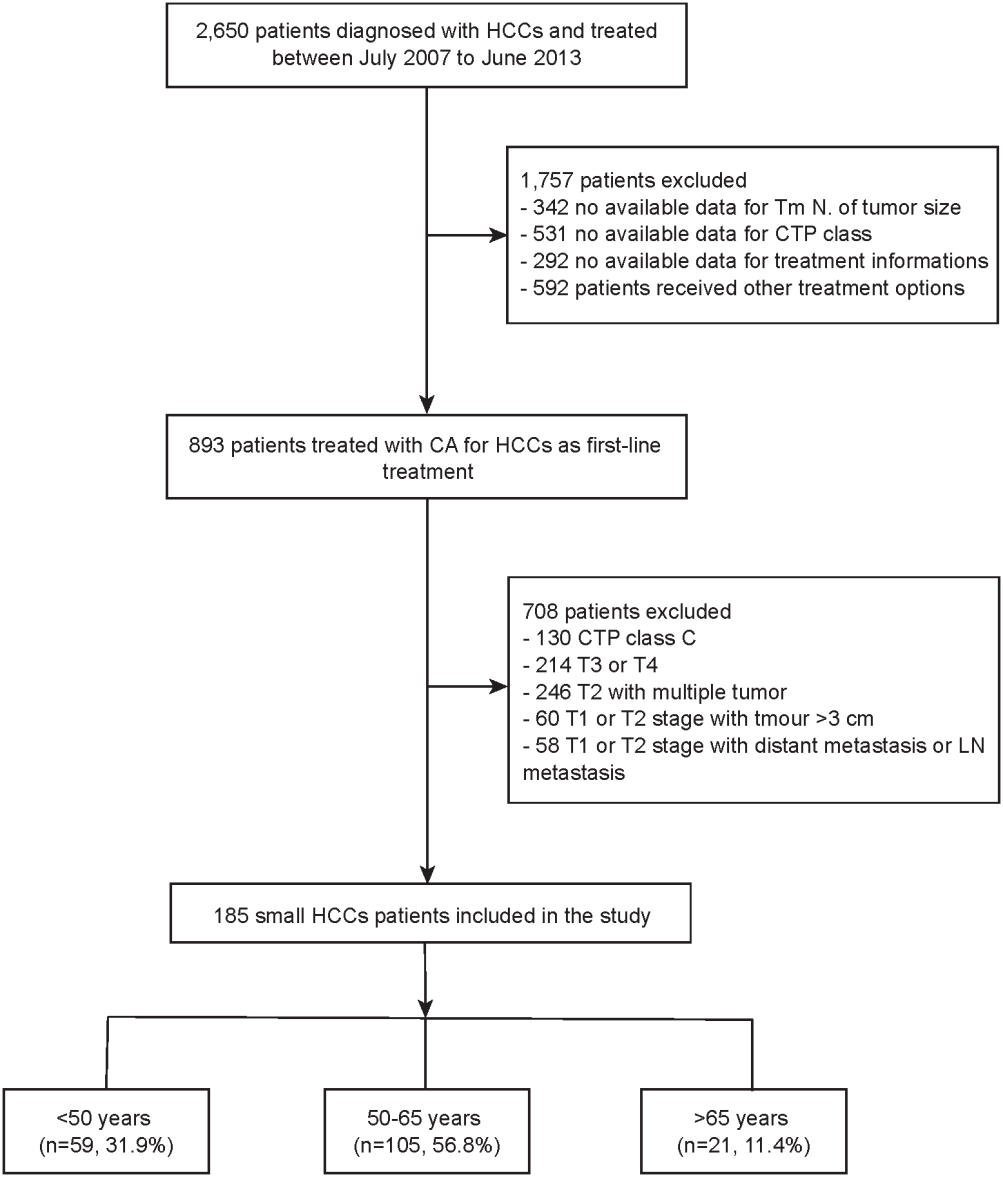
The flowchart of enrolled patients.

This retrospective study was approved by the medical ethics committee of the Fifth Medical Center of Chinese PLA General Hospital, China. Written informed consent was obtained from each patient in the study. From July 2007 to June 2013, 185 patients with a clinical diagnosis of small HCC who were treated with percutaneous CRYO were included in this study (**Figure 1**). According to the Barcelona Clinic Liver Cancer (BCLC) System, small HCC is defined as very early or early stage HCC (11, 12). Inclusion criteria of this study were as follows: (1) small HCC confirmed by imaging or pathological examination; (2) ineligible for surgical resection or liver transplantation; without evidence of vascular invasion, bile duct invasion, or extrahepatic metastasis; (3) preoperative CT/MRI imaging scans, laboratory test records and survival information are available; and (4) CRYO was an initial treatment. The exclusion criteria are following: (1) recurrent small HCC; (2) with severe comorbidities that cannot endure treatment; (3) severe coagulation disturbance; (4) patients who met inclusion criteria but declined to participate in the study or follow up; (5) patients who preferred to receive surgical resection or liver transplantation treatment or other therapies.

### Percutaneous argon-helium CA procedures

The argon-helium based EndoCare system (EndoCare, Irvine, CA, USA) was applied to perform CRYO (13–15). Various sizes of cryoprobes were used (2 or 3 mm in diameter). The area to be frozen included both the entire tumor area and at least 5-10 mm of paraneoplastic liver tissue outside the tumor area. After local anesthesia, the cryoprobe was inserted into the tumor by a percutaneous approach using CT guidance. The cryoprobe is advanced under guidance until it reaches the distal edge of the target lesion. A dual freeze-thaw cycle consists of 20 minutes of freezing, 10 minutes of thawing, and another 15 minutes of freezing. After removal of the probe, all tracts were packed through the intrathecal guide with Surgicel (Johnson & Johnson, Arlington, TX, USA) to control bleeding, and then the intrathecal guide was removed.

### Follow-up

The follow-up protocol including routine physical examination, laboratory tests and prothrombin time, and contrast-enhanced imaging including CT or MRI. All patients were followed up every three months in the first year and twice a year thereafter to detect tumor recurrence.

### Variable collection and definition

Demographic information, serologic biochemical test outcomes, alpha-fetoprotein (AFP) and types of underlying chronic liver disease were collected. Liver function was assessed by Child-Pugh grade. Physical condition was assessed by Eastern Cooperative Oncology Group (ECOG) score. The short-term outcomes included the number of patients with complete ablation, the number of patients with postoperative minor complications and major complications. Long-term outcomes included the number of deaths, the number of tumor-related deaths, the number of patients with tumor progression, the mean overall survival (OS) and progression-free survival (PFS). Complete ablation was defined as tumor tissue completely covered by the ablation area and there was no enhancement in the ablation area during the initial 1-month CT and MRI follow-up (13). Minor complications were defined as adverse events leading to minor consequences, including pain, fever, and bleeding from the needle tract. Major complications were defined as adverse events leading to serious consequences, including hepatic rupture and bleeding, spontaneous peritonitis, and death.

### Statistical analysis

Patients were divided into three groups according to age distribution: group 1, <50 years, group 2, 50–65 years, and group 3, >65 years. To reduce the effect of bias and confounding variables, a 1:1 propensity score matching with a caliper of 0.05 was used to match group 3 with the other groups. The continuous variables are expressed as the mean and standard deviation and the categorical variables are expressed as the frequency or percentage. To compare differences between groups, continuous variables were analyzed by the Mann-Whitney U test and Kruskal-Wallis test when non-normally distributed, and categorical variables were analyzed by the Chi-Square test or Fisher exact test as appropriate. OS and PFS were analyzed by the Kaplan-Meier (KM) method and compared by the log-rank test. The rate of LTP, distant recurrence, and OS was calculated using the Kaplan-Meier method. Univariate and multivariate Cox analyses were performed to determine the potential relationships between variables and prognostic outcomes. A two-sided P value <0.05 was considered statistically significant. Packages R software (http://www.R-project.org; The R Foundation) was used to perform all statistical analyses.

## Results

### Patients included and baseline characteristics

The detailed information of 185 patients with small HCC are summarized in **Table 1**. There are 144 men and 41 women, with a mean age of 53.8 ± 9.7 (range, 22-75). Of these patients, 166 patients (89.7%) were HBsAg (surface antigen of the hepatitis B virus) positive, 25 patients (13.5%) were anti-HCV (hepatitis C virus) positive, 6 patients (3.2%) were both HBsAg positive and anti HCV positive, and 2 patients (1.1%) for other reasons (alcoholic). Regarding liver function, 164 patients (88.6%) were Child–Pugh Class A and 21 patients (11.4%) were Child–Pugh B. The mean length of postoperative follow-up was 45.2 months.

**Table 1:** Baseline characteristics of included patients.

Variable	Group 1 (*n*=59)	Group 2 (*n*=105)	Group 3 (*n*=21)	*P* value
Age (years)	42.98 ± 5.87	56.78 ± 4.45	69.62 ± 3.11	<0.01
Sex (male)	45 (76.3%)	87 (82.9%)	15 (71.4%)	0.361
BMI				0.989
<18.5	6 (10.2%)	13 (12.4%)	2 (9.5%)	
18.5-24.9	42 (71.2%)	72 (68.6%)	16 (76.2%)	
>25	11 (18.6%)	20 (19.0%)	3 (14.3%)	
Hypertension	21 (35.6%)	45 (42.9%)	7 (33.3%)	0.547
Diabetes mellitus	5 (8.5%)	13 (12.4%)	3 (14.3%)	0.668
Cardiovascular disease	26 (44.1%)	71 (67.6%)	9 (42.9%)	<0.01
Respiratory disease	4 (6.8%)	16 (15.2%)	8 (38.1%)	<0.01
Cerebrovascular disease	1 (1.7%)	5 (4.8%)	3 (14.3%)	0.084
Tumor size (cm)	2.26 ± 0.68	2.13 ± 0.78	2.26 ± 0.82	0.513
AFP (mg/L)	454.36 ± 1033.52	224.90 ± 734.08	86.99 ± 139.05	0.386
Platelet count (×10^9^/L)	101.69 ± 67.92	105.04 ± 57.43	112.96 ± 46.40	0.342
Albumin (g/L)	36.46 ± 5.53	37.17 ± 5.49	37.29 ± 4.51	0.885
Total bilirubin (μmol/L)	19.23 ± 12.40	20.14 ± 17.05	20.01 ± 14.04	0.996
Underlying chronic liver disease				0.042
HBV infection	56 (94.9%)	91 (86.7%)	19 (90.5%)	
HCV infection	3 (5.1%)	15 (14.3%)	7 (33.3%)	
Other	1 (1.7%)	1 (1.0%)	0 (0.0%)	
Child-Pugh grade				0.495
A	51 (86.4%)	95 (90.5%)	18 (85.7%)	
B	8 (13.6%)	10 (9.5%)	3 (14.3%)	
ECOG score				0.863
0	0 (0.0%)	1 (1.0%)	0 (0.0%)	
1	54 (91.5%)	90 (85.7%)	18 (85.7%)	
2	5 (8.5%)	14 (13.3%)	3 (14.3%)	

BMI, Body mass index; AFP, alpha-fetoprotein; HBV, hepatitis B virus; HCV, hepatitis C virus; ECOG, Eastern Cooperative Oncology Group.

### Comparison of baseline characteristics

The number of patients included in the three groups was as follows: 59 patients (31.9%) in group 1, 105 patients (56.8%) in group 2, and 21 patients (11.4%) in group 3. Significant difference between groups was observed in the baseline variables of underlying chronic liver disease (P=0.042) (**Table 1**).

### Comparison of prognosis outcomes before propensity score matching

The short-term and long-term outcomes in the three age groups are listed in the **Table 2**. With regard to the short-term outcomes, the complete ablation was obtained in 56 (94.9%), 90 (85.7%) and 19 (90.5%) patients in groups 1-3 (P=0.189), respectively. The patients who developed minor complications were 31 (52.5%), 33 (31.4%) and 6 (28.6%) (P=0.022), respectively. The patients who developed major complications were 1 (1.7%), 7 (6.7%) and 0 (0.0%) (P=0.304), respectively.

**Table 2:** Prognostic outcomes for groups 1–3.

Variable	Group 1 (*n*=59)	Group 2 (*n*=105)	Group 3 (*n*=21)	*P* value
Short-term outcomes				
Complete ablation	56 (94.9%)	90 (85.7%)	19 (90.5%)	0.189
Minor complications	31 (52.5%)	33 (31.4%)	6 (28.6%)	0.022
Major complications	1 (1.7%)	7 (6.7%)	0 (0.0%)	0.304
Long-term outcomes				
Death	6 (10.2%)	6 (5.7%)	1 (4.8%)	0.585
Tumor-specific death	1 (1.7%)	1 (1.0%)	0 (0.0%)	1
OS	40.99 ± 31.67	47.05 ± 30.49	47.73 ± 29.21	0.303
Tumor progression	25 (42.4%)	53 (50.5%)	9 (42.9%)	0.560
PFS	22.06 ± 20.22	23.15 ± 19.33	33.22 ± 29.30	0.337

OS overall survival, PFS progression-free survival.

**Figure 2:**
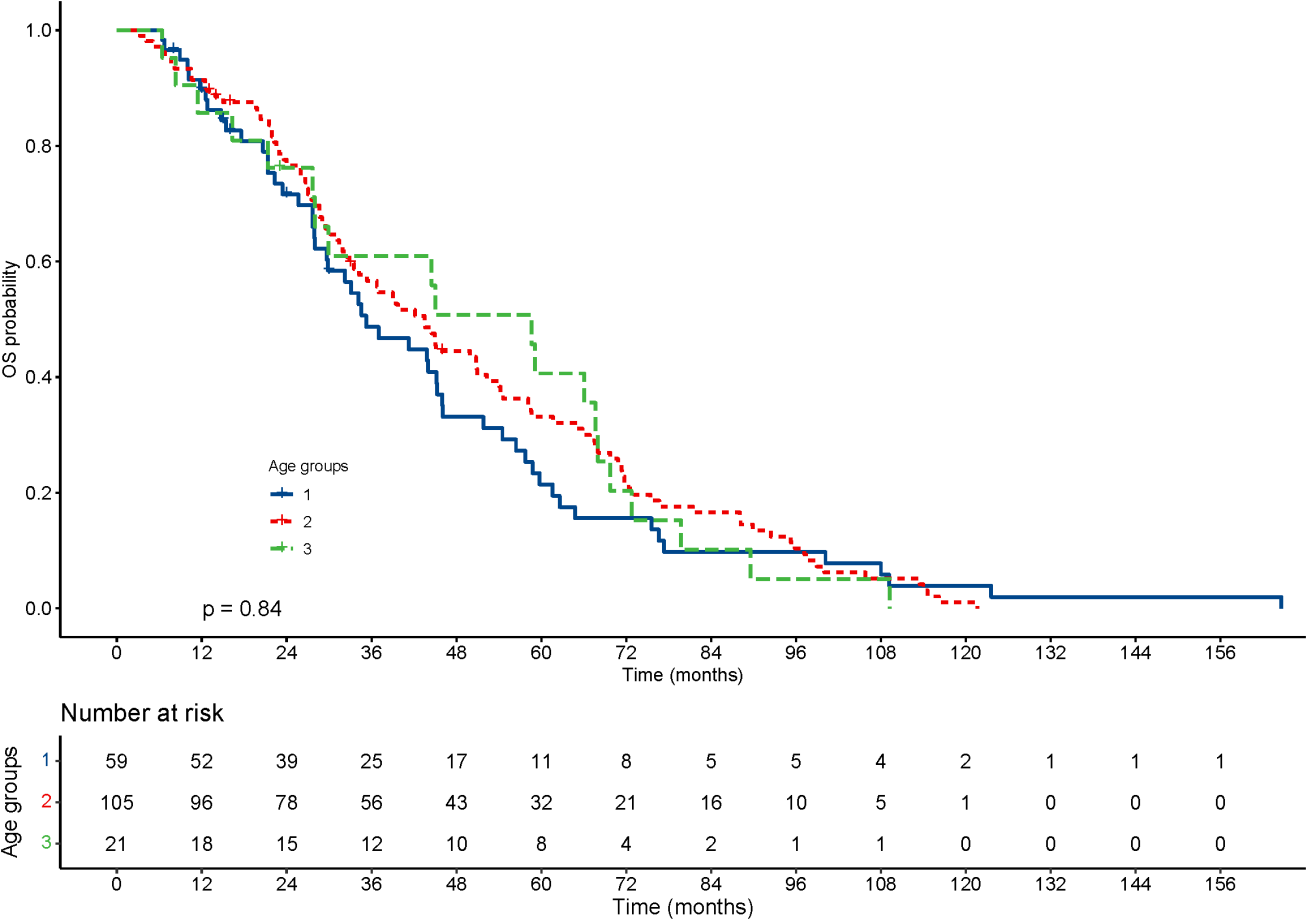
Kaplan–Meier curves for overall survival (OS) of patients with small hepatocellular carcinomas after cryoablation. The log-rank test showed no significant difference between the four groups (P=0.84).

**Figure 3:**
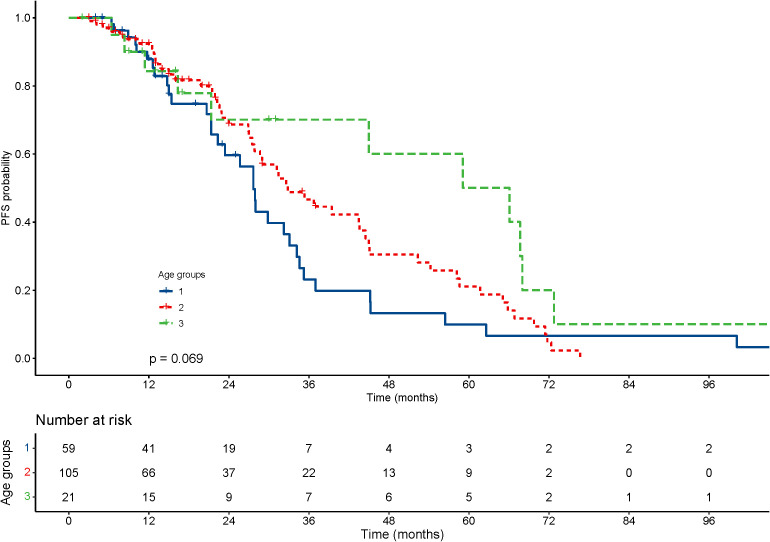
Kaplan–Meier curves for progression-free survival (PFS) of patients with small hepatocellular carcinomas after cryoablation. The log-rank test showed no significant difference between the four groups (P =0.069).

With regard to the long-term outcomes, the number of deaths in groups 1-3 was 6 (10.2%), 6 (5.7%) and 1 (4.8%) (P=0.585), respectively. The number of tumor-specific deaths in groups 1-3 was 1 (1.7%), 1 (1.0%) and 0 (0.0%) (P=0.1), respectively. The OS was 40.99 ± 31.67, 47.05 ± 30.49 and 47.73 ± 29.21 months in group 1-3 (P=0.303), respectively. The rates of progression were 42.4%, 50.5% and 42.9% in group 1-3 (P=0.560), respectively. The PFS was 22.06 ± 20.22, 23.15 ± 19.33 and 33.22 ± 29.30 months in group 1-3 (P=0.337), respectively. The KM curve of OS is shown in **Figure 2** and the log rank test found no significant differences (P=0.84) among the age groups. The KM curve of PFS is shown in **Figure 3** and the log rank test found no significant differences (P=0.069) among the age groups.

### Comparison of prognosis outcomes after propensity score matching

To further investigate the effect of age on prognosis, we used the group 3 as the elderly group and the first two groups as the younger group and compared these two groups. To reduce the effect of bias and confounding variables, including sex, BMI, the size of the tumors, AFP, albumin, total bilirubin, underlying chronic liver disease, comorbid conditions, Child Pugh grade and ECOG score, the propensity score was used to match comparable patients to obtain the younger group for comparison. The median age of younger group was 51.9 ± 9.1 years. The baseline characteristics of the patients before and after matching are shown in **Table 3**. A comparison of short-term and long-term outcomes before and after matching is shown in **Table 4**.

**Table 3:** Baseline characteristics of Group younger (≤65 years) and Group elderly (>65 years) before and after matching.

Variable	Younger (*n*=164)	Elderly (*n*=21)	*P* value	Younger (*n*=19)	Elderly (*n*=19)	*P* value
Age (years)	51.8 ± 8.3	69.6 ± 3.1	<0.001	51.9 ± 9.1	69.4 ± 3.0	<0.001
Sex (male)	129 (78.7%)	15 (71.4%)	0.637	16 (84.2%)	15 (78.9%)	1
BMI			0.933			0.864
<18.5	19 (11.6%)	2 (9.5%)		2 (10.5%)	2 (10.5%)	
18.5-24.9	114 (69.5%)	16 (76.2%)		13 (68.4%)	15 (78.9%)	
>25	31 (18.9%)	3 (14.3%)		4 (21.1%)	2 (10.5%)	
Hypertension	66 (40.2%)	7 (33.3%)	0.709	6 (31.6%)	6 (31.6%)	1
Diabetes mellitus	18 (11.0%)	3 (14.3%)	0.713	4 (21.1%)	3 (15.8%)	1
Cardiovascular disease	97 (40.2%)	9 (42.9%)	0.235	5 (26.3%)	9 (47.4%)	0.313
Respiratory disease	20 (12.2%)	8 (38.1%)	<0.01	8 (42.1%)	7 (36.8%)	1
Cerebrovascular disease	6 (3.7%)	3 (14.3%)	0.068	1 (5.3%)	3 (15.8%)	0.604
Tumor size (cm)	2.2 ± 0.7	2.3 ± 0.8	0.639	2.1 ± 0.9	2.3 ± 0.9	0.573
AFP (mg/L)	307.4 ± 858.0	87.0 ± 139.1	0.003	347.3 ± 716.6	83.9 ± 141.4	0.125
Platelet count (×10^9^/L)	103.8 ± 61.2	113.0 ± 46.4	0.511	82.9 ± 37.3	114.4 ± 45.3	0.025
Albumin (g/L)	36.9 ± 5.5	37.3 ± 4.5	0.767	36.7 ± 5.0	37.6 ± 4.5	0.588
Total bilirubin (μmol/L)	19.8 ± 15.5	20.0 ± 14.0	0.955	19.2 ± 1.7	18.9 ± 12.9	0.941
Underlying chronic liver disease			0.082			0.191
HBV infection	147 (89.6%)	19 (90.5%)		18 (94.7%)	17 (89.5%)	
HCV infection	18 (11.0%)	7 (33.3%)		1 (5.3%)	5 (26.3%)	
Other	2 (1.2%)	0 (0.0%)		0 (0.0%)	0 (0.0%)	
Child-Pugh grade			0.74			1
A	146 (89.0%)	18 (85.7%)		17 (89.5%)	17 (89.5%)	
B	18 (11.0%)	3 (14.3%)		2 (10.5%)	2 (10.5%)	
ECOG score			0.725			0.105
0	1 (0.6%)	0 (0.0%)		0 (0.0%)	0 (0.0%)	
1	144 (87.8%)	18 (85.7%)		15 (78.9%)	18 (94.7%)	
2	19 (11.6%)	3 (14.3%)		4 (21.1%)	1 (5.3%)	

BMI, Body mass index; AFP, alpha-fetoprotein; HBV, hepatitis B virus; HCV, hepatitis C virus; ECOG, Eastern Cooperative Oncology Group.

**Table 4:** Prognostic outcomes before and after matching.

Variable	Younger (*n*=164)	Elderly (*n*=21)	*P* value	Younger (*n*=19)	Elderly (*n*=19)	*P* value
Short-term outcomes						
Complete ablation	146 (89.0%)	19 (90.5%)	1.000	18 (94.7%)	17 (89.5%)	1.000
Minor complications	64 (39.0%)	6 (28.6%)	0.352	10 (52.6%)	5 (26.3%)	0.097
Major complications	8 (4.9%)	0 (0.0%)	0.642	0 (0.0%)	0 (0.0%)	NA
Long-term outcomes						
Death	12 (7.3%)	1 (4.8%)	1.000	1 (5.3%)	1 (5.3%)	1.000
Tumor-specific death	7 (4.1%)	0 (0.0%)	0.813	0 (0.0%)	0 (0.0%)	NA
OS	44.9 ± 30.0	47.7 ± 29.2	0.689	77.6 ± 38.6	46.6 ± 28.8	0.008
Tumor progression	78 (47.6%)	9 (42.9%)	0.684	13 (68.4%)	7 (36.8%)	0.050
PFS	22.8 ± 19.6	33.2 ± 29.3	0.126	25.6 ± 25.5	35.0 ± 29.9	0.303

OS, overall survival; PFS, progression-free survival; NA, not available.

With regard to the short-term outcomes, the number of complete ablations in group younger and older was 146 (89.0%) and 19 (90.5%) (P=1.000), respectively. The number of patients with minor complications was 64 (39.0%) and 6 (28.6%) (P=0.352), respectively. The number of patients with major complications was 8 (4.9%) and 0 (0.0%) (P=0.642), respectively. After matching, there was still no significant difference in the number of patients with complete ablation (P=1.000) and number of patients with minor complications (P=0.097).

**Figure 4:**
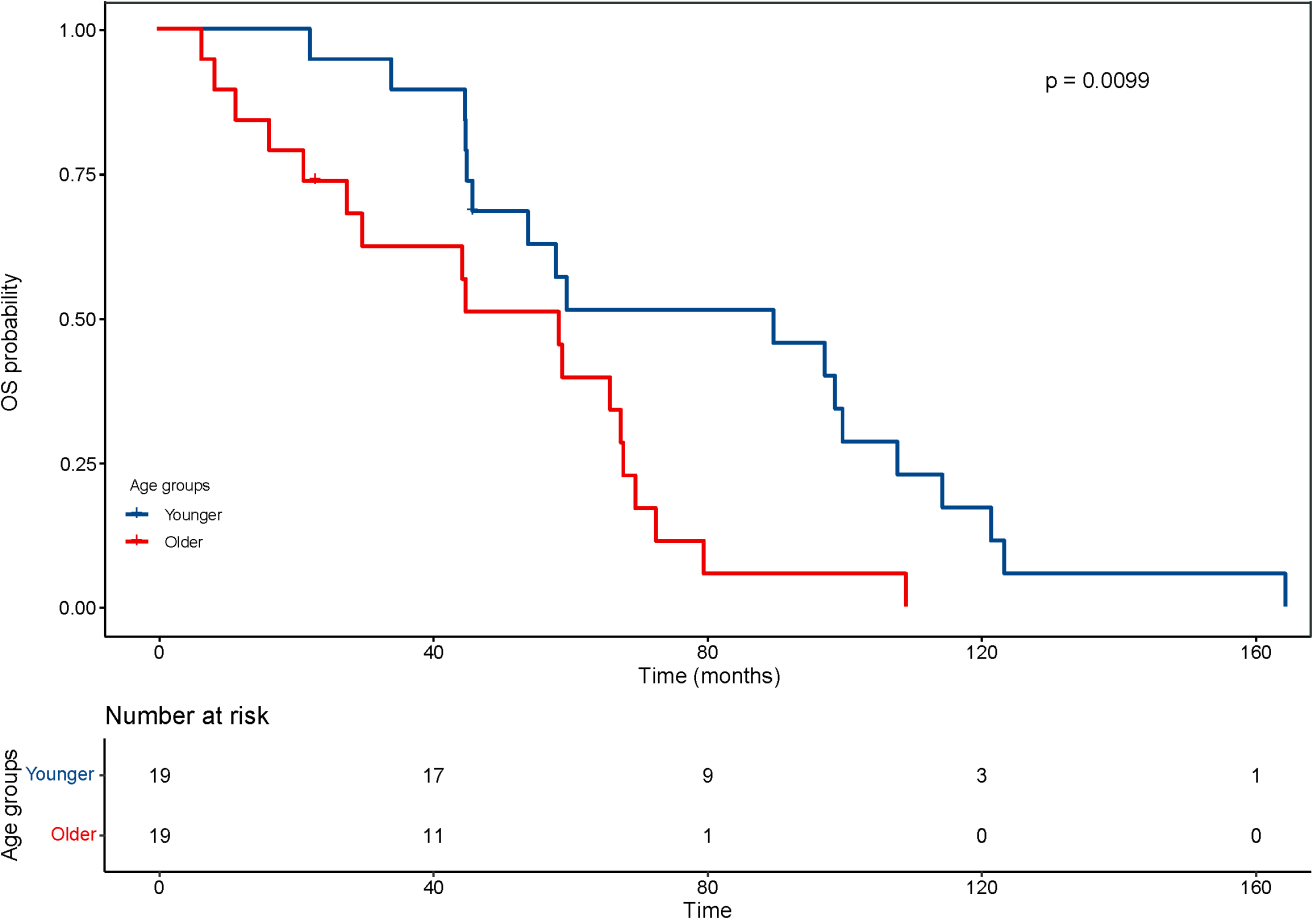
Kaplan–Meier curves for overall survival (OS) of patients with small hepatocellular carcinomas after cryoablation. The log-rank test showed significant difference between the elderly and young groups (P <0.01).

**Figure 5:**
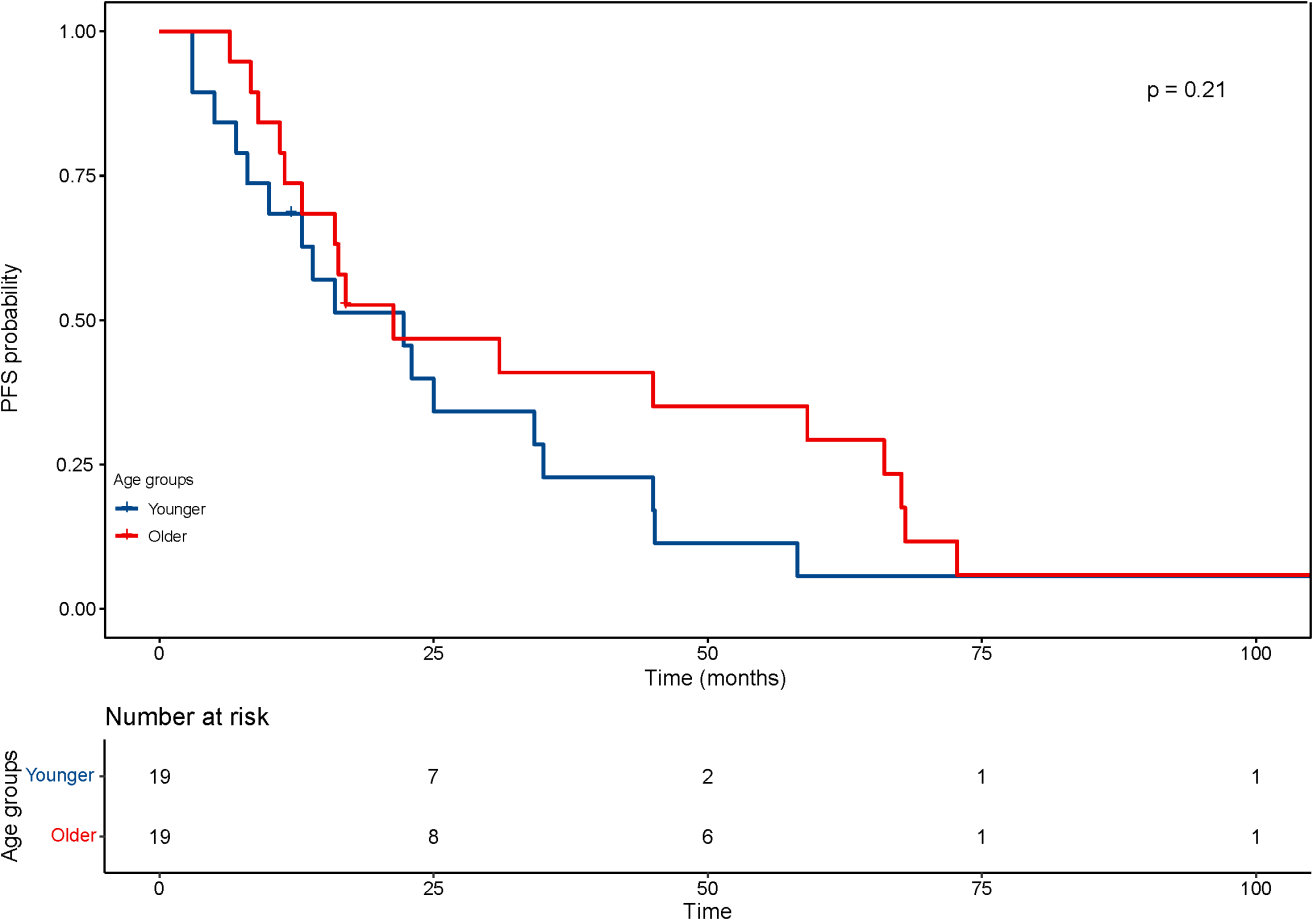
Kaplan–Meier curves for progression-free survival (PFS) of patients with small hepatocellular carcinomas after cryoablation. The log-rank test showed no significant difference between the four groups (P =0.21).

With regard to the long-term outcomes, the number of deaths in group younger and older was 12 (7.3%) and 1 (4.8%) (P=1.000), respectively. The number of tumor-specific death was 7 (4.1%) and 0 (0.0%) (P=0.813), respectively. The OS was 44.9 ± 30.0 and 47.73 ± 29.21 months (P=0.689), respectively. The rates of progression were 47.6% and 42.9% (P=0.684), respectively. The PFS was 22.8 ± 19.6 and 33.22 ± 29.30 months (P=0.126), respectively. After matching, the OS was 77.6 ± 38.6 and 46.6 ± 28.8 months (P=0.008), respectively. The KM curve of OS is shown in **Figure 4** and the log rank test found significant differences (P=0.010). The rates of progression were 68.4% and 36.8% (P=0.050), respectively. The PFS was 25.6 ± 25.5 and 35.0 ± 29.9 months (P=0.303), respectively. The KM curve of PFS is shown in **Figure 5** and the log rank test found no significant differences (P=0.210) among the age groups.

### Analysis of risk factors for prognosis outcomes

The univariate analysis revealed that the use of alcohol (HR=3.3, P=0.045), Child-Pugh grade (HR=8.5, P<0.001), platelet count (HR=0.98, P=0.034), albumin (HR=0.83, P<0.001) and total bilirubin (HR=1, P=0.017) were predictors associated with overall survival. The multivariate analysis showed that the Child-Pugh grade (HR=3.1, P<0.001), albumin (HR=0.85, P=0.004) and TBIL (HR=1, P=0.024) were the independent prognostic factor (**Table 5**).

**Table 5:** Univariate and multivariate analyses of factors associated with overall survival (OS).

Univariate						Multivariate		
		95% CI				95% CI		
	HR	Lower	Upper	*P* value	HR	Lower	Upper	*P* value
Age, years	0.97	0.92	1	0.300				
Sex, male	0.29	0.038	2.2	0.230				
BMI	0.99	0.37	2.7	0.980				
Family history of HCV	1.3	0.3	6.1	0.700				
Family history of HBV	1.1	0.62	2	0.710				
Alcohol	3.3	1	11	0.045				
Smoke	1.6	0.5	4.8	0.450				
Diabetes	0.69	0.09	5.3	0.720				
Tumor size, cm	0.82	0.39	1.8	0.620				
Child-Pugh grade	8.5	3	24	<0.001	3.1	1.5	19	<0.001
ECOG score	1	0.31	3.3	0.990				
AFP, mg/L	1	1	1	0.550				
Platelet count (×10^9^/L)	0.98	0.97	1	0.034				
Albumin, g/L	0.83	0.75	0.91	<0.001	0.85	0.77	0.95	0.004
Total bilirubin, μmol/L	1	1	1	0.017	1	1	1.1	0.024

BMI, Body mass index; AFP, alpha-fetoprotein; HBV, hepatitis B virus; HCV, hepatitis C virus; ECOG, Eastern Cooperative Oncology Group.

## Discussion

Numerous age-related changes in hepatic structure and function have been described, including changes in the size of hepatocytes and changes in the mitochondria and endoplasmic reticulum inside the hepatocytes. Some animal experiments have also confirmed the declined function and regeneration rate of the liver in aged animals. These age-related changes have important clinical implications with regard to treatment options for small hepatocellular carcinoma. However, some studies suggest that age should not be a determining factor in such clinical decisions. Some elderly patients exhibit decreased adaptive hepatic responsiveness, characterized by decreased hepatic clearance of drugs and increased rates of adverse drug reactions, while maintaining liver function within the normal range. In the present study, the OS after CA decreased with ageing, with a mean of 77.6 ± 38.6 months in the younger patient group (under 65 years) and 46.6 ± 28.8 months in the elderly patient group (over 65 years). However, the PFS after CA was not significantly associated with age.

The findings of this study may have implications for clinical practice. Clinicians are concerned about referring elderly patients with small HCC for surgery because of minimal benefit from open surgery at advanced age. In this clinical scenario, minimally invasive treatment modalities may usually be considered. The study by Zhang et al. (16) found that elderly patients with HCC, even if associated with more comorbidities, may achieve similar prognostic outcomes after microwave ablation (MWA) as younger patients. However, similar studies have not been performed extensively in elderly patients undergoing cryoablation. Cryoablation is an extremely effective treatment modality, more research on the cost-effectiveness of cryoablation needs to be conducted to gain more insight into the most suitable surgical population.

In the current study, we compared the baseline characteristics and prognosis of patients in different age groups and found some differences between these groups. We observed significant differences in the distribution of hepatitis virus types and tumor-related mortality. Among them, with increasing age, the probability of liver cancer is higher in HCV-infected patients, but lower in HBV-infected patients, which is similar to the results of other studies (17, 18). The reason behind this phenomenon may be due to the older age of HCV-infected patients than HBV-infected patients. We consider that elderly patients are a particular population with unique characteristics. Therefore, after matching patients in the older and younger groups by propensity score matching, we found that patients in the older group had shorter overall survival and lower rates of tumor progression. In addition, there were no significant differences in short-term postoperative outcomes and PFS between the two age groups after matching, indicating that the safety and progression-free survival of CA did not vary with age, further implying that the survival outcomes of the older group were not worse than those of the younger patients, as shown in **Table 4**. These findings suggest that older patients can benefit equally from CA compared to younger patients with similar liver function and tumor burden.

Over the decades, many biomarkers have been shown to be effective predictors of liver tumor recurrence and prognosis, including Child-Pugh grade, alpha-fetoprotein (AFP), alanine aminotransferase (ALT), albumin (ALB), and TBIL (19–21). Among them, the Child-Pugh and Model for End-Stage Liver Disease (MELD) scores are well-known prognostic tools for liver function and have been widely used in the prognosis of patients with liver disease. The Child-Pugh grade is used as a frequently used tool to assess liver function and predict postoperative outcomes, including subjective variables such as ascites, TBIL and encephalopathy. Several previous studies have found a strong sensitivity of the Child-Pugh grade in predicting the prognosis of various treatment modalities for HCC. Zhang et al. showed that the Child-Pugh grade can be used as an independent risk factor to predict the prognosis of microwave ablation procedures for HCC. Huang et al. found that the Child-Pugh grade performed better in predicting the prognosis of HCC patients undergoing hepatectomy and was more accurate than the ALBI score. In the present study, Child-Pugh grade and TBIL were shown by Cox regression analysis to be independent risk factors for predicting prognosis in patients with small HCC, and these results were similar to those of previous studies.

The Glasgow prognostic score, which includes serum albumin levels and C-reactive protein, is a powerful prognostic assessment tool for a variety of malignancies (22, 23). Among them, serum albumin levels have been shown to play an essential role in the prognosis of HCC. Several researchers found that albumin gene expression levels and mRNA levels were significantly lower in liver tumor tissues compared to normal human liver tissues (24, 25). A study by Bağırsakçı et al. (26) showed that lower albumin levels were associated with larger tumor volumes and higher AFP levels. Also, they found that the adding of albumin to HCC cell lines significantly inhibited the growth of tumor cells. Similar to these studies, in the current study, we also found that higher serum albumin levels were a protective factor for the prognosis of small HCC. The results of the present study may contribute to further clarification of HCC-related prognostic parameters.

There are some limitations to this study. First, the most significant limitation of this study was the relatively small number of patients >65 years old included, which may have diminished the statistical power of the results. Future studies need to further expand the cohort of patients of advanced age to elicit more reliable results. Second, this was a single-center study with a relatively limited sample size included. This may have affected the results of the study. Therefore, the sample should be further expanded in future studies to validate the findings of this study. Third, the applicable population for the results of this study needs further discussion. In the Chinese population, most HCC are caused by hepatitis B virus, however, in the European and American populations, most HCC are caused by hepatitis C virus and alcoholic. Therefore, more multicenter and multiethnic studies need to be conducted to validate the findings of this study. Fourth, the efficacy of CA is related to the surgical technique. Surgical techniques of different physicians among different centers may result in inconsistent prognostic outcomes.

In conclusion, this study shows that the elderly population can achieve acceptable prognostic outcomes with PFS times similar to those of younger people after treatment with CA. Also, we found that Child-Pugh grade and TBIL were independent risk factors for poor prognosis, while higher serum albumin levels was a protective factor. Our findings further demonstrate the suitability of this type of surgery for CA in elderly patients and provide a clinical reference for the indication of surgery.

## Data availability statement

The raw data supporting the conclusions of this article will be made available by the authors, without undue reservation.

## Ethics statement

The studies involving human participants were reviewed and approved by the medical ethics committee of the Fifth Medical Center of Chinese PLA General Hospital. The patients/participants provided their written informed consent to participate in this study. Written informed consent was obtained from the individual(s) for the publication of any potentially identifiable images or data included in this article.

## Author contributions

Concept and design: YY and ZZ. Data analysis: HZ, MX, JS, HK, XG, WZ, XC, BY, YC, ZD, JH. Writing and critical analysis: All. All authors contributed to the article and approved the submitted version.

## References

[1] Forner A Reig M Bruix J. Hepatocellular carcinoma. Lancet. (2018) 391(10127):1301–14. doi: 10.1016/s0140-6736(18)30010-2 29307467

[2] Hartke J Johnson M Ghabril M. The diagnosis and treatment of hepatocellular carcinoma. Semin Diagn Pathol (2017) 34(2):153–9. doi: 10.1053/j.semdp.2016.12.011 28108047

[3] Nault JC Sutter O Nahon P Ganne-Carrié N Séror O. Percutaneous treatment of hepatocellular carcinoma: State of the art and innovations. J Hepatol (2018) 68(4):783–97. doi: 10.1016/j.jhep.2017.10.004 29031662

[4] Zhu F Rhim H. Thermal ablation for hepatocellular carcinoma: what's new in 2019. Chin Clin Oncol (2019) 8(6):58. doi: 10.21037/cco.2019.11.03 31968982

[5] Izzo F Granata V Grassi R Fusco R Palaia R Delrio P. Radiofrequency ablation and microwave ablation in liver tumors: An update. Oncologist. (2019) 24(10):e990–e1005. doi: 10.1634/theoncologist.2018-0337 31217342 PMC6795153

[6] Tajiri K Shimizu Y. Liver physiology and liver diseases in the elderly. World J Gastroenterol (2013) 19(46):8459–67. doi: 10.3748/wjg.v19.i46.8459 PMC3870491 24379563

[7] Pibiri M. Liver regeneration in aged mice: new insights. Aging (Albany NY). (2018) 10(8):1801–24. doi: 10.18632/aging.101524 PMC6128415 30157472

[8] Xu F Hua C Tautenhahn HM Dirsch O Dahmen U. The role of autophagy for the regeneration of the aging liver. Int J Mol Sci (2020) 21(10):3606. doi: 10.3390/ijms21103606 PMC7279469 32443776

[9] Schmucker DL. Age-related changes in liver structure and function: Implications for disease? Exp Gerontol. (2005) 40(8-9):650–9. doi: 10.1016/j.exger.2005.06.009 16102930

[10] Rimassa L Personeni N Czauderna C Foerster F Galle P. Systemic treatment of HCC in special populations. J Hepatol (2021) 74(4):931–43. doi: 10.1016/j.jhep.2020.11.026 33248171

[11] Reig M Forner A Rimola J Ferrer-Fabrega J Burrel M Garcia-Criado A. BCLC strategy for prognosis prediction and treatment recommendation: The 2022 update. J Hepatol (2022) 76(3):681–93. doi: 10.1016/j.jhep.2021.11.018 PMC8866082 34801630

[12] Xu XL Liu XD Liang M Luo BM. Radiofrequency ablation versus hepatic resection for small hepatocellular carcinoma: Systematic review of randomized controlled trials with meta-analysis and trial sequential analysis. Radiology. (2018) 287(2):461–72. doi: 10.1148/radiol.2017162756 29135366

[13] Zhang W Gao X Sun J Cheng J Hu Y Dong Z. Percutaneous argon-helium cryoablation for small hepatocellular carcinoma located adjacent to a major organ or viscus: A retrospective study of 92 patients at a single center. Med Sci Monit (2021) 27:e931473. doi: 10.12659/msm.931473 34385410 PMC8369936

[14] Yang W An Y Li Q Liu C Zhu B Huang Q. Co-Ablation versus cryoablation for the treatment of stage III-IV non-small cell lung cancer: A prospective, noninferiority, randomized, controlled trial (RCT). Thorac Cancer. (2021) 12(4):475–83. doi: 10.1111/1759-7714.13779 PMC7882381 33319493

[15] Luo J Dong Z Xie H Zhang W An L Yu Z. Efficacy and safety of percutaneous cryoablation for elderly patients with small hepatocellular carcinoma: A prospective multicenter study. Liver Int (2022) 42(4):918–29. doi: 10.1111/liv.15169 35065003

[16] Zhang YX Zhang XH Yu XL Han ZY Yu J Liu FY. Prognosis of microwave ablation for hepatocellular carcinoma: does age make a difference? Int J Hyperthermia. (2020) 37(1):688–95. doi: 10.1080/02656736.2020.1778198 32558602

[17] Cohen MJ Bloom AI Barak O Klimov A Nesher T Shouval D. Trans-arterial chemo-embolization is safe and effective for very elderly patients with hepatocellular carcinoma. World J Gastroenterol (2013) 19(16):2521–8. doi: 10.3748/wjg.v19.i16.2521 PMC3646143 23674854

[18] Sagnelli E Macera M Russo A Coppola N Sagnelli C. Epidemiological and etiological variations in hepatocellular carcinoma. Infection. (2020) 48(1):7–17. doi: 10.1007/s15010-019-01345-y 31347138

[19] Lee SC Tan HT Chung MC. Prognostic biomarkers for prediction of recurrence of hepatocellular carcinoma: current status and future prospects. World J Gastroenterol (2014) 20(12):3112–24. doi: 10.3748/wjg.v20.i12.3112 PMC3964383 24696598

[20] Heimbach JK Kulik LM Finn RS Sirlin CB Abecassis MM Roberts LR. AASLD guidelines for the treatment of hepatocellular carcinoma. Hepatology. (2018) 67(1):358–80. doi: 10.1002/hep.29086 28130846

[21] Wang NY Wang C Li W Wang GJ Cui GZ He H. Prognostic value of serum AFP, AFP-L3, and GP73 in monitoring short-term treatment response and recurrence of hepatocellular carcinoma after radiofrequency ablation. Asian Pac J Cancer Prev (2014) 15(4):1539–44. doi: 10.7314/apjcp.2014.15.4.1539 24641364

[22] McMillan DC. The systemic inflammation-based Glasgow prognostic score: a decade of experience in patients with cancer. Cancer Treat Rev (2013) 39(5):534–40. doi: 10.1016/j.ctrv.2012.08.003 22995477

[23] Ando K Sakamoto S Saito S Maimaiti M Imamura Y Sazuka T. Prognostic value of high-sensitivity modified Glasgow prognostic score in castration-resistant prostate cancer patients who received docetaxel. Cancers (Basel). (2021) 13(4):773. doi: 10.3390/cancers13040773 PMC7918602 33673284

[24] Wu GX Lin YM Zhou TH Gao H Pei G. Significant down-regulation of alpha-albumin in human hepatoma and its implication. Cancer Lett (2000) 160(2):229–36. doi: 10.1016/s0304-3835(00)00589-9 11053653

[25] Ljubimova JY Petrovic LM Wilson SE Geller SA Demetriou AA. Expression of HGF, its receptor c-met, c-myc, and albumin in cirrhotic and neoplastic human liver tissue. J Histochem Cytochem (1997) 45(1):79–87. doi: 10.1177/002215549704500111 9010472

[26] Bağırsakçı E Şahin E Atabey N Erdal E Guerra V Carr BI. Role of albumin in growth inhibition in hepatocellular carcinoma. Oncology. (2017) 93(2):136–42. doi: 10.1159/000471807 28486226

